# A new method for experimental characterisation of scattered radiation in 64-slice CT scanner

**DOI:** 10.2349/biij.6.1.e3

**Published:** 2010-01-01

**Authors:** A Akbarzadeh, MR Ay, H Ghadiri, S Sarkar, H Zaidi

**Affiliations:** 1 Department of Medical Physics and Biomedical Engineering, University of Medical Sciences, Tehran, Iran; 2 Research Center for Science and Technology in Medicine, Tehran University of Medical Sciences, Tehran, Iran; 3 Research Institute for Nuclear Medicine, Tehran University of Medical Sciences, Tehran, Iran; 4 Department of Medical Physics, Iran University of Medical Sciences, Tehran, Iran; 5 Division of Nuclear Medicine, Geneva University Hospital, CH-1211 Geneva 4, Switzerland; 6 Geneva Neuroscience Center, Geneva University, CH-1205 Geneva, Switzerland

**Keywords:** Scatter, SPR, CT, tube voltage

## Abstract

**Purpose:**

The consummate 64-slice CT scanner that spawns a new generation of non-invasive diagnostic tool, however revolutionary, brings with it the incidental by-product that is scattered radiation. The extended detector aperture capability in the 64-slcie CT scanner allows the effects of scattered radiation to be more pronounced and therefore demands that the magnitude and spatial distribution of scatter component be addressed during the imaging process. To this end, corrective algorithms need to be formulated on a basis of a precise understanding of scatter distribution. Relative to a 64-slice CT scanner, here now a unique solution is based upon dedicated blockers operative within various detector rows, calculating scatter profiles and scatter to primary ratios (SPR).

**Materials and methods:**

A single dimension blocker array was installed beneath the collimator, and the extrapolated shadow area on the detectors revealed the scatter radiation after exposure. The experiment was conducted using a 64-slice CT scanner manufactured by GE Healthcare Technologies.

**Results:**

Variables such as tube voltage, phantom size and phantom-off centring on the scatter profile and the SPR was measured using the dedicated blocker method introduced above. When tube voltage is increased from 80kVp to 140kVp in a 21.5 cm water phantom, the SPR is found to reduce from 219.9 to 39.9 respectively.

**Conclusion:**

The method developed within this study is applicable to any measurement and is direct with minimal complexity.

## INTRODUCTION

X-ray computed tomography (CT) scans are inherently associated with unwanted by-products of error that have a deteriorating impact on image quality. If the impairment of the image quality is substantial, a corrective algorithm aimed at providing some redress may be called for or alternatively, modification of the geometric schematics of the scanner may be in order. One such side effect that is inherent to CT imaging is the noise brought about by scatter radiation. Resulting artefacts may be disposed of successfully with careful consideration of the scanner design, or it may be corrected for during the imaging procedure.

As a result of scatter radiation in CT imaging, data is corrupted and cupping errors thereby affect the reconstructed images. For state of the art CT scanners incorporating cone-beam configurations with extended detector aperture, the result is exaggerated. These multi-detector scanners are far more prone to these artefacts than their cousins, the fan-beam CT scanners.

While scanner design remains at the heart of any available solution, in order to resolve these anomalies, the magnitude and spatial distribution of scattered radiation collected by a CT scanner needs to be quantified accurately. From this premise scanner design can be modified to reflect the optimal geometric configuration demanded by the task, and also extensive corrective techniques can be applied to reduce remnant scatter radiation.

The incidence of scatter radiation has attracted the remedy of mathematical modelling, specifically Monte Carlo simulations for fan-beam and cone-beam configurations. Indeed, the research to date on scatter radiation distribution in fan-beam configurations uses single blockers [3,4,5] or Monte Carlo simulations [6,7,8] to deduce a conclusion.

Here is put forward, a revolutionary method of incorporating dedicated blockers to measure the scatter profile and the scatter-to-primary ratio (SPR) with precision. This is applicable to all detector rows within a 64-slice CT scanner.

As this method measures scatter radiation in multiple detector rows, it improves on the limitations of the single measurement points in previous methods.

## MATERIALS AND METHODS

### Acquisition system

A 64-slice Light Speed VCT scanner, manufactured by GE Healthcare Technologies (Waukesha, WI) equipped with Highlight (Y_2_Gd_2_O_3_:Eu) ceramic scintillators was the CT scanner of choice for this study. The source to isocentre distance on this CT scanner is 540mm, while the source to detector distance is 950mm. Among 64 rows each with 888 active patient elements and 24 reference elements, 58,368 individual elements of 0.625mm thickness at isocentre are distributed. Complementing the scanner is a Performix Pro Anode Grounded Metal-Ceramic Tube Unit using a 56° fan angle, a 7° target angle, and a minimum inherent filtration of 3.25mm Al and 0.1mm Cu at 140kVp.

### Phantoms

In order to measure the scattered radiation profile and the SPR, a water-filled cylindrical phantom with 215mm internal diameter and a 6 mm thick Plexiglas wall was constructed, together with a uniform polypropylene cylindrical phantom with a 300mm diameter. The purpose of the polypropylene phantom is to recreate clinical conditions present when the subject is obese.

### Lead blocker array

While it has its limitations, the usual method of calculating SPR is by placing a small lead blocker on top of the phantom. Measurement of scattered radiation is possible by monitoring the x-ray intensity from behind the phantom to the underside of the blocker, but this is only possible at one point.

**Figure 1:**
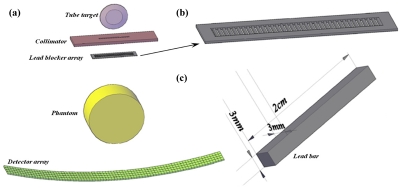
(a) Schematic drawing of setup utilised for scatter profile estimation. (b) Lead blocker array. (c) Lead bar and its dimensions.

However, this study suggests placing a lead blocker array after the collimator (Figure 1a). This array is constituted of 20 lead bars of 3mm thickness. The attenuation coefficient for lead is 3.32cm^2^/gr and with its density being 11.3 gr/cm^3^, the linear attenuation coefficient for lead is 37.52 cm^-1^. The transmission capability of 3mm of lead is approximately 1.24394x10^-5^.

When the array is placed under the second collimator it is 160mm under the source. With the economy of only one irradiation, not only are the scattered and primary radiation levels in numerous detector channels able to be measured, but also the SPR at various locations.

### Methods

The following measurements were collected without the usual bow-tie filter that normally resides within the collimator box.

The lead blocker is placed between the collimator and the phantom during tube exposure with different voltage (kVp) and current (mA) settings.

In order to eliminate the scatter produced by the lead blocker itself, irradiation is performed twice; once to obtain the precise measurement of the scatter that occurs without the phantom in position, and once with the phantom in place. The scatter from the lead blocker array is then compensated for.

The raw data from the CT scanner is transferred from the scanner’s database to a PC for remote processing. The CT scanner has 64 detector rows and 912 detector channels in each row. The target file contains the entire 912 x 64 detector readings.

Due to the fact that scanner software invariably applies certain calibration factors, these factors must be identified and applied inversely in order to obtain the untainted data. The scanner software used in this study is the Light Speed VCT Scanner’s Data Acquisition System, which reduces high value readings from the detectors to within a 16 digit range to successfully compress data into a manageable file size.

To implement this processing of the data that is extracted from the CT scanner, a program was written in Matlab 7.4, to read the binary data file, apply the inverse of the calibration factors, and obtain the untainted data output in a 2-dimensional matrix (912 x 64).

This matrix attributes values to reflect the amount of radiation on each detector, and is divided into two sections; a shadowed area reflecting the particular detector channels that were within the coverage of the lead blocker array (invariably recording scatter radiation if any), and also an unshadowed area that represented the detector channels that remained exposed (recording total radiation both primary and scatter radiation).

The algorithm governing the conversion of these data first interpolated the total radiation profiles and also the scatter radiation profiles for all detector channels, and then found primary radiation by deduction of the latter from the former. SPR ratios were then easily calculated for each detector channel.

## Results

### Scatter profiles

**Figure 2:**
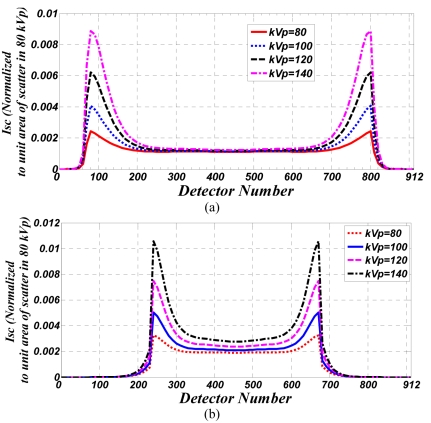
Intensity of scattered radiation (Isc) in all 912 channels of 32^nd^ detector row at different tube voltages and 100 mA tubes current. for (a) cylindrical water phantom (b) cylindrical Polypropylene phantom.

Figure 2(a) shows the scatter profile for detector row 32, which is the central row in the CT scanner, after the cylindrical water phantom was irradiated for 3 seconds. Various x-ray tube voltages were applied, with a constant tube current of 100mA.

Figure 2(b) shows the scatter profile, also for detector row 32, after the cylindrical Polypropylene phantom with 300mm diameter was irradiated for 3seconds. Various x-ray tube voltages were again applied, also with a constant tube current of 100mA.

The scanner software has normalised these measurements.

### Primary radiation

**Figure 3:**
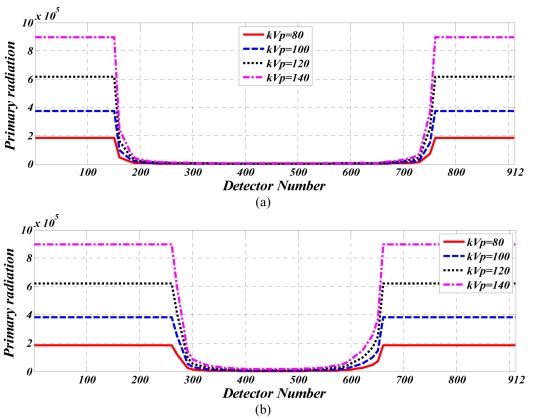
Primary radiation recorded on all 912 channels of 32^nd^ detector row at different tube voltages and 100 mA tube current. for (a) cylindrical water phantom (b) cylindrical Polypropylene phantom.

Figure 3(a) shows the primary radiation recorded on row 32, after irradiation of the cylindrical water phantom (diameter 215mm), again at varying x-ray tube voltages and a constant tube current of 100mA.

Figure 3(b) shows the primary radiation recorded on row 32, after irradiation of the cylindrical Polypropylene phantom (diameter 300mm), under the same conditions.

These values have had the calibration coefficients inversely applied and as such produce the raw detector readings without normalisation.

### Scatter to primary ratio

This ratio is a useful qualitative measurement to investigate the extent that scatter photons pollute projection data. The SPR is a function of phantom size, phantom material, tube voltage and phantom off-centring.

**Figure 4:**
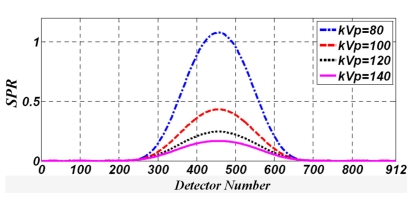
Calculated SPR profiles in all 912 channels of the 32^nd^ detector row for the water phantom at different tube voltages and 100 mA tube current.

Figure 4 shows the SPR for the cylindrical water phantom at various tube voltages and a tube current of 100mA.

**Figure 5:**
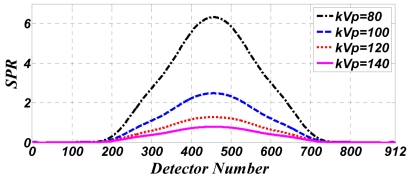
Calculated SPR profiles in all 912 channels of the 32^nd^ detector row for Polypropylene phantom at different tube voltages and 200 mA tube current.

Figure 5 shows the SPR for the Polypropylene phantom, also at various tube voltages and yet a 200 mA tube current. Again, this phantom is used to recreate clinical conditions present when the subject is obese.

### Integrated SPR

The integrated SPR is the sum of the SPR with respect to all the detectors in a particular row.

**Figure 6:**
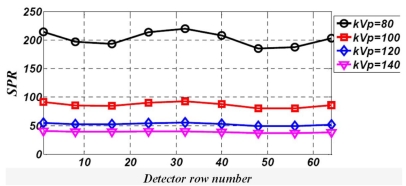
Integrated SPR for different detector rows in 64-slice CT scanner obtained for the water phantom irradiated at different tube voltages.

Figure 6 shows the integrated SPR for the 64 detector rows of the scanner when the cylindrical water phantom is irradiated at varying tube voltages.

The central row (no. 32) has integrated SPR values of 219.5, 92.5, 55.2 and 39.9 for tube voltages of 80, 100, 120 and 140 kVp respectively.

**Figure 7:**
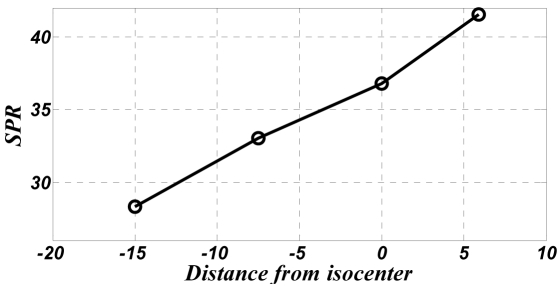
Integrated SPR in the water phantom calculated for different distances between the phantom and detectors (positive: toward the detector and negative: toward the x-ray tube) for tube current of 100mA and 120 kVp tube voltage.

Figure 7 shows the resultant SPR with respect to a change in distance between the phantom and the particular detector (here the 32^nd^ detector row). The positive and negative values indicate the phantom being moved towards, and away from the detector respectively.

It is conclusive that the greater the distance between the phantom and the detector, the lower the SPR. Both the tube voltage and the tube current were constant at 120 kVp and 100mA respectively.

## DISCUSSION

The results of this experiment using a lead blocker array are congruent with current research on the subject, and as such the method introduced is able to be applied to measure scatter radiation distribution and SPR’s.

The two peaks displayed in Figure 2(a) and 2(b) are more likely a result of the Compton effect (that occurs when photons interact with matter), and to a less extent due to the larger attenuation length of photons scattered from the phantom. Since the phantom covers this area, the lower amount of scatter radiation in the centre of the row is due to photons either being absorbed before the Compton Effect or due to attenuation after the Compton Effect.

Figures 4 and 5 show the highest SPR for the lowest tube voltage applied which is 80kVp.

While the chance of a Compton effect is increased with an increase in tube voltage (as Figures 2 and 3 indicate), primary radiation i.e. primary photons increase at a greater rate than the number of scattered photons, when tube voltage is increased. For this reason, the SPR decreases when tube voltage is increased.

Arguably, SPR decreases proportionally to an increase in the distance between the phantom and the detector [1,5,6,8]. Known as the air-gap effect, this is obviously attributable to the reduction in the number of scattered photons that the detector records.

## CONCLUSION

A lead block array was used to measure scatter radiation distribution and SPR in a 64-slice CT scanner, but this revolutionary method is able to be adapted to other multi-slice CT scanners.

The method introduced is practical and able to be applied in the acquisition of any related measurement, and can be used to accurately extrapolate data to produce a scatter profile.

Designers of CT scanners are able to use the accurate deduction of magnitude and spatial distribution of scattered radiation that this method provides, and in doing so improve the geometry of scanners to achieve the optimal corrective algorithms to reduce scatter radiation.

Considering the commercial introduction of 256-slice [9] and 320-slice CT scanners, advances in this respect are timely will be highly appreciated.
